# Increased arterial stiffness in patients with end-stage osteoarthritis: a case-control study

**DOI:** 10.1186/s12891-016-1201-x

**Published:** 2016-08-11

**Authors:** Kaspar Tootsi, Aare Märtson, Mihkel Zilmer, Kaido Paapstel, Jaak Kals

**Affiliations:** 1 Department of Traumatology and Orthopaedics, University of Tartu, Puusepa street 8, Tartu, Estonia; 2 Clinic of Traumatology and Orthopaedics, Tartu University Hospital, Tartu, Estonia; 3 Institute of Biomedicine and Translational Medicine, Centre of Excellence for Genomics and Translational Medicine, University of Tartu, Tartu, Estonia; 4 Department of Vascular Surgery, Tartu University Hospital, Tartu, Estonia; 5 Department of Surgery, University of Tartu, Tartu, Estonia

**Keywords:** Osteoarthritis, Arterial stiffness, Pulse wave velocity, Cardiovascular risk, Inflammation

## Abstract

**Background:**

Both osteoarthritis (OA) and cardiovascular diseases (CVD) are prevalent conditions which often co-exist. Vascular involvement in the pathogenesis of these diseases, as well as increased cardiovascular risk in OA patients give occasion to investigate arterial stiffness in OA. The aim of this study was to establish associations between OA and arterial stiffness.

**Methods:**

The characteristics of arterial stiffness were measured with Sphygmocor and HDI devices in 48 patients (age 63 ± 7 years (mean ± SD)) with end-stage OA awaiting knee and hip replacement and in 49 age and gender matched controls (61 ± 7 years). Independent Student’s *t*-test or the Mann-Whitney *U* test was used to compare means between the groups. Correlation between variables was determined using Pearson’s or Spearman’s correlation analysis and stepwise multiple regression analysis.

**Results:**

Carotid-femoral pulse wave velocity (car-fem PWV) was increased in the patients with OA compared to the controls (9.6 ± 2.4 and 8.4 ± 1.9 m/s, *p* = 0.015 respectively). High-sensitivity C-reactive protein and white blood cells count were significantly higher in the OA patients compared with the controls (1.80 ± 1.10 and 1.48 ± 1.32 mg/l, *p* = 0.042; 6.5 ± 1.5 and 5.6 ± 1.9 10^9^/l, *p* = 0.001 respectively). In multiple regression analysis age (*p* < 0.001), mean arterial blood pressure (*p* = <0.001) and OA status (*p* = 0.029) were found to be independent predictors of car-fem PWV.

**Conclusions:**

This study showed that patients with OA had increased aortic stiffness compared to non-OA controls. The potential link between arterial stiffening and OA suggests that vascular alterations are involved in OA pathogenesis and could be responsible for increased cardiovascular risk in end-stage OA patients.

## Background

Osteoarthritis (OA) is a chronic progressive disease that affects the whole joint: cartilage, bone, joint capsule, synovial membrane, ligaments and periarticular muscles. Due to overweight and aging populations, the disease has a huge and growing socioeconomic impact [1]. Knee OA alone affects more than 250 million people worldwide [2]. OA patients have increased cardiovascular risk [3–5]. In a prospective study, Barbour et al. [4] found that radiographic hip OA increased cardiovascular mortality by 25 % and all-cause mortality by 43 %. Similarly, in a prospective longitudinal study, Rahman et al. [6] found OA to be associated with higher risk of ischemic heart disease. However, it remains to be elucidated whether OA and cardiovascular diseases (CVD) are causally related, or whether this association is due to shared risk factors.

Arterial stiffness is an established marker of increased cardiovascular risk [7, 8] but it has rarely been studied in OA patients. There is evidence that hand OA, a more systemic form of OA, is related to increased arterial stiffness [9]. In contrast, a study of 206 patients with symptomatic knee OA found no association between arterial stiffness and bone marrow lesions, a surrogate marker for OA [10]. However, the data about possible interactions between OA and arterial stiffness are contradictory and need further research.

Many mechanisms responsible for OA pathogenesis are also involved in development of vascular damage. Common risk factors include aging, obesity and limited physical activity, as well as high exposure to non-steroidal anti-inflammatory drugs that increase CVD risk. Also, excessive oxidative stress has been found to occur in patients with OA [11, 12]. Oxidative stress has a significant role in development of OA by promoting inflammation, cellular senescence and apoptosis [13]. Chronic low-grade inflammation is crucial in deterioration of OA joints [14, 15]. Analogously, arterial stiffness and endothelial dysfunction have also been found to be associated with oxidative stress [16, 17] and inflammation [18, 19]. Obesity is considered a risk factor for both OA and CVD. Adipose tissue produces cytokines and adipokines that damage cartilage and arteries [20, 21]. Thus, there is a potential pathogenetic link between OA and increased arterial stiffness via inflammation and oxidative stress. The aim of this present study was to elucidate, besides conventional cardiovascular risk factors, also the role of arterial stiffness in patients with OA.

## Methods

### Study population

Patients, with primary knee or hip OA, who were eligible for total joint replacement at Tartu University Hospital, Estonia in 2014–2015, were included. The OA was assessed using the American College of Rheumatology’s criteria for knee and hip OA [22, 23]. Our study includes both knee and hip OA and focuses on systemic changes that accompany OA. Subjects with pre-existing hypertension were included. Patients with any acute or chronic inflammatory disease, diabetes, coronary artery disease, cardiac arrhythmias or known valve pathology, peripheral atherosclerotic disease, malignancies or renal insufficiency (eGFR < 60 ml/min/1.73 m^2^) were excluded.

Age and sex matched controls with no prior joint problems were selected from family medical practices. The family medical practices are in the catchment area of Tartu University Hospital. Selection was made, using the group matching strategy, on the basis of clinical examination and not on plain radiography, which is known to be insensitive to early OA [24] and correlates poorly with patients’ disability (pain, function) and disease severity [25]. The exclusion criteria for the control group were (based on interviews, clinical examinations and blood tests): any concomitant acute or chronic inflammatory disease, a visit to their family practitioner due to hip or knee joint complaints, any persistent knee or hip joint pain, diabetes, coronary artery disease, cardiac arrhythmia, cerebrovascular or peripheral artery disease, malignancies and renal insufficiency.

The study was conducted according to the Helsinki declaration and received approval from the Ethics Committee of the University of Tartu. Written informed consent was obtained from all participants.

### Study protocol

Blood samples were collected from the antecubital fossa between 07:00–11:00 after an overnight fast and abstinence from tobacco. Study participants’ height, weight, waist and hip circumference were recorded and joint-specific evaluation was performed. The Harris Hip Score (HHS) was used for hip joint evaluation and the Hospital for Special Surgery (HSS) Knee Score was used for knee joint evaluation. Both of these tests have a scale of 0–100, where 100 is the best possible outcome. After 15 min of rest in a temperature controlled quiet room in a supine position, participants’ blood pressure and pulse wave velocity (PWV) were recorded and pulse wave analysis was made. All pulse wave parameters were measured at least twice and the average was calculated.

### Hemodynamic measurements

Blood pressure was measured using a digital oscillometric device (A&D UA-767; A&D Company Ltd., Tokyo, Japan) and the mean of three readings was recorded. Mean arterial pressure was obtained by integration of the radial pressure waveform using the SphygmoCor software (SCOR Px, 7.0; AtCor Medical, Sydney, Australia). Arterial stiffness was measured using pulse wave analysis and PWV. Left radial artery waveforms were recorded with a high fidelity micromanometer (SPT-301BH; Millar Instruments, Tx., USA). Corresponding ascending aortic waveforms were then generated using a transfer function to calculate central hemodynamics and the augmentation index (AIx). The AIx was corrected for a heart rate of 75 beats per minute. Carotid-femoral (car-fem) and carotid-radial (car-rad) PWV were measured by sequentially recording ECG-gated carotid and femoral or radial artery waveforms using a Sphygmocor device (SphygmoCor; AtCor Medical, Sydney, Australia) and the foot-to-foot method [26]. The pulse waveform was recorded from the right radial artery with a Cardiovascular Profiling Instrument (HDI/Pulse Wave CR-2000; Hypertension Diagnostics Inc., Eagan, USA) which measures large (C1) and small (C2) artery elasticity.

### Laboratory analysis

Plasma glucose, complete blood count, triglycerides, total cholesterol, LDL-cholesterol and HDL-cholesterol, creatinine and urea were measured by standard laboratory methods, using certified assays, in a local clinical laboratory.

### Radiographic evaluation

Standard weight-bearing antero-posterior knee and hip joint radiographs were taken in the OA group, no radiographs were taken in the control group. Osteoarthritis was assessed according to the Kellgren-Lawrence grading system, where: 0 = no changes; 1 = doubtful joint space narrowing; 2 = definite osteophytes and doubtful joint space narrowing; 3 = definite ostephytes, joint space narrowing, sclerosis and possible deformity; 4 = marked joint space narrowing, large osteophytes, severe sclerosis and definite bone deformity [27]. The radiographs were evaluated independently by two raters blinded to the clinical data and a consensus score was used.

### Statistical analysis

The statistical analysis software SPSS 22.0 (SPSS Inc., Chicago IL, USA) for windows was used for data analysis. The Shapiro- Wilk test was used to ascertain whether variables were normally distributed. Continuous variables are presented as means with ± standard deviation. Dichotomous variables are presented as prevalence in number. A two tailed Student’s *t*-test was used for detecting differences between the groups for normally distributed data and logarithmic transformation or Mann-Whitney *U* test was used for non-parametric data. Pearson’s correlation coefficient and Spearman’s rho were used to identify associations between continuous variables within the study groups. A Chi-square test or Fischer’s exact test was used to compare group proportions where appropriate. Multiple linear regression analyses with the use of a forward and backward stepwise variable selection procedure, wereperformed to investigate the independent associations between variables. The authors set the goal to detect a difference of 1 m/s in car-fem PWV. The standard deviation for the study group was estimated at 2.0. Statistical power calculations showed that group size had to be *n* = 41 for either group with an Alpha error level of 5 % and a Beta error level of 20 %. In order to further minimize Beta error level the number of subjects was increased to 48 + 49. Interrater reliability analysis, with the use of intraclass correlation coefficient (ICC), was performed to determine consistency among the raters of radiographic severity of OA.

## Results

The study population consisted of a total of 97 participants of whom 48 were with hip or knee osteoarthritis and 49 were healthy volunteers. The general anthropometric characteristics of the participants are presented in Table 1. Both groups had approximately the same age, BMI, male to female ratio, waist to hip ratio and number of active smokers. The OA group included 23 subjects and the control group included 19 subjects with hypertension, of whom 10 in either group were using hypertension medications. There was a clear difference in HHS and HSS Knee Score between the groups: both were significantly higher in the control group. The median radiographic Kellgren-Lawrence score in the OA group was 3 (range 2–4). The ICC for the raters was found to be ICC = 0.73, 95 % CI (0.53-0.85).

**Table 1:** General parameters of osteoarthritis patients and controls

Variable	Osteoarthritis (*n = 48*)	Controls (*n = 49*)	*P*-value
Age (years)	63 ± 7	61 ± 7	0.115
Male/Female (n)	25/23	24/25	0.760
Height (cm)	1.71 ± 0.10	1.72 ± 0.10	0.508
Weight (kg)	80.8 ± 12.6	78.8 ± 15.1	0.487
BMI (kg/m^2^)	27.6 ± 3.0	26.4 ± 3.4	0.068
Waist circumference (cm)	96 ± 10	92 ± 12	0.046
Hip circumference (cm)	104 ± 7	102 ± 6	0.387
W/H- ratio	0.92 ± 0.08	0.90 ± 0.10	0.137
Current smoker (*n*, %)	10 (14)	7 (21)	0.430
Hypertension (%)	48	39	0.416
Involved joint (n)			
hip	30	–	
knee	18	–	
Harris Hip score	39 (33–47)	100 (100–100)	<0.001
HSS Knee score	62 (49–65)	100 (100–100)	<0.001

The values are expressed as means ± s.d. or medians (with interquartile range)

*Abbreviations*: *BMI* body-mass index, *W/H-ratio* waist to hip ratio, *HSS* Hospital for Special Surgery

### Hemodynamics

There was a significant difference between the OA patients and the controls in central arterial stiffness parameter. Car-fem PWV was significantly higher in the OA group compared to controls (Table 2). Central blood pressure and peripheral blood pressure did not differ significantly between the study groups. There was no significant difference in pulse pressure amplification, or in C1 and C2 levels between the OA and non-OA groups. There was significant difference in car-fem PWV between the hip and the knee OA patients (9.2 ± 2.0 and 10.6 ± 2.7 m/s, *p* = 0.042).

**Table 2:** Hemodynamic and laboratory analysis parameters of osteoarthritis patients and healthy controls

Variable	Osteoarthritis patients (*n* = 48)	Controls (*n* = 49)	*p*-value
Peripheral SBP (mm Hg)	133.6 ± 17.8	128.3 ± 18.9	0.158
Peripheral DBP (mm Hg)	80.8 ± 7.5	78.9 ± 9.0	0.269
AIx@HR75 (%)	25.0 ± 8.1	22.8 ± 9.4	0.213
PP amplification (%)	123 ± 11	124 ± 13	0.879
Central SBP (mm Hg)	125.2 ± 17.0	120.5 ± 18.8	0.205
Central DBP (mm Hg)	81.5 ± 8.2	79.8 ± 9.3	0.373
Mean pressure (mm Hg)	99.6 ± 10.9	96.6 ± 12.2	0.209
Heart rate (bpm)	64.8 ± 7.1	63.2 ± 7.3	0.279
car-fem PWV (m/s)	9.6 ± 2.4	8.4 ± 1.9	0.015
car-rad PWV (m/s)	8.7 ± 1.1	8.4 ± 1.1	0.235
C1 (ml/mmHg × 100)	12.5 ± 4.2	12.7 ± 3.5	0.371
C2 (ml/mmHg × 100)	4.5 ± 2.9	5.6 ± 4.3	0.315
Triglycerides (mmol/l)	1.7 ± 0.8	1.4 ± 0.7	0.021
LDL- cholesterol (mmol/l)	4.1 ± 1.0	3.8 ± 1.0	0.234
HDL- cholesterol (mmol/l)	1.5 ± 0.5	1.7 ± 0.5	0.830
Total cholesterol (mmol/l)	6.0 ± 1.1	6.0 ± 1.3	0.264
Glucose (mmol/l)	5.8 ± 0.7	5.7 ± 0.4	0.566
hsCRP (mg/l)	1.80 ± 1.10	1.48 ± 1.32	0.042
Urea (mmol/l)	5.9 ± 2.0	5.3 ± 1.3	0.171
eGFR (ml/mg/1.73 m^2^)	83 ± 20	82 ± 12	0.434
White blood cells (10^9^/L)	6.5 ± 1.5	5.6 ± 1.9	0.001
Plateletes (10^9^/L)	243 ± 52	222 ± 39	0.053

The values are expressed as means ± s.d

*Abbreviations*: *SBP* systolic blood pressure, *DBP* diastolic blood pressure, *AIx@HR75* augmentation index corrected for a heart rate of 75 beats per minute, *AP* aortic augmentation, *car-fem PWV* carotid to femoral pulse wave velocity, *car-rad PWV* carotid to radial pulse wave velocity, *C1* large artery elasticity index, *C2* small artery elasticity index, *LDL* low-density lipoprotein, *HDL* high-density lipoprotein, *hs-CRP* high-sensitivity C-reactive protein, *eGFR* estimated glomerular filtration rate

### Laboratory analysis

The results of laboratory analysis are presented in Table 2. Triglyceride level was significantly higher in the OA patients compared to the controls. The levels of LDL and HDL-cholesterol, glucose, urea and eGFR did not differ between the groups. High-sensitivity C-reactive protein (hsCRP) and white blood cells count were significantly higher in the OA group and the difference in platelet count was of borderline significance.

**Fig. 1:**
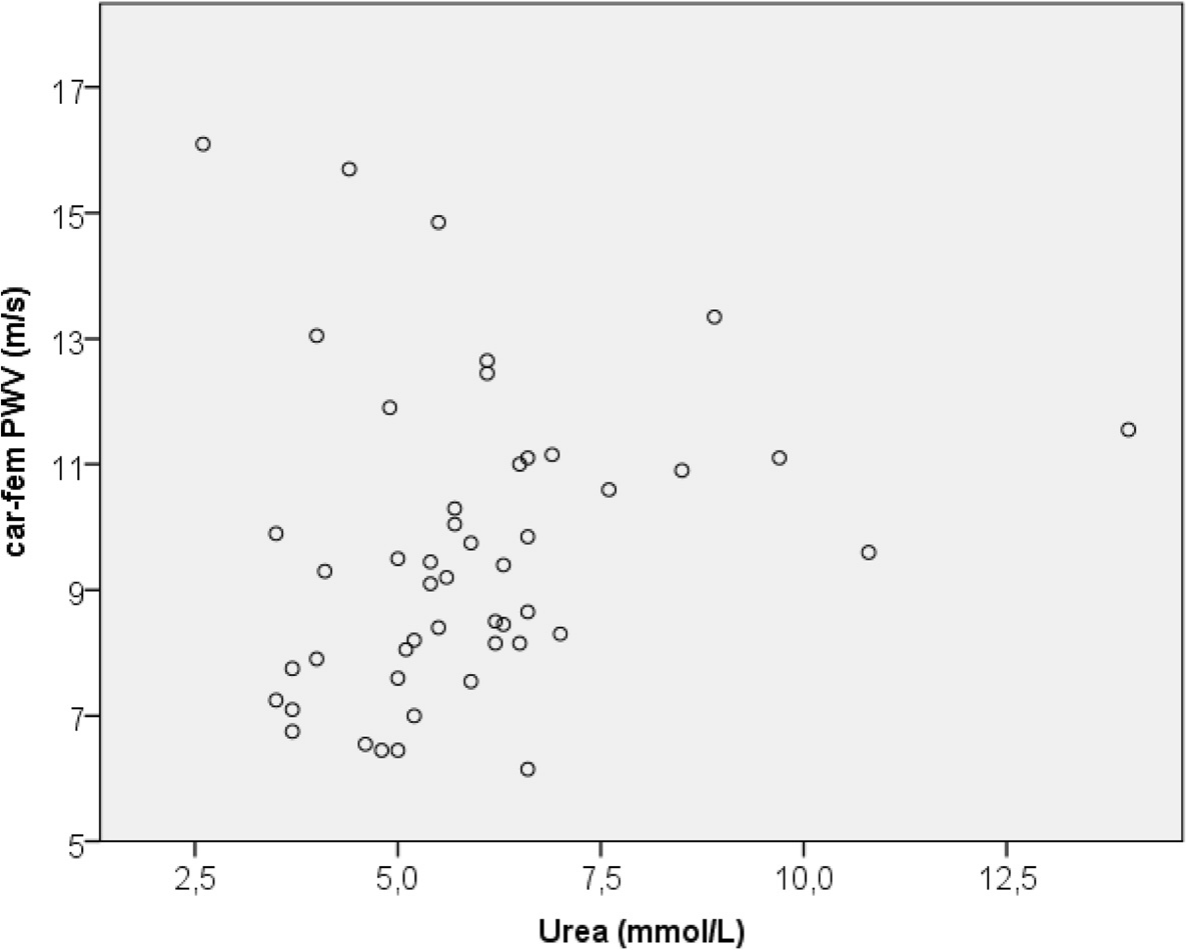
The association between car-fem PWV and serum urea level (rho = 0.290, *p* = 0.046)

**Fig. 2:**
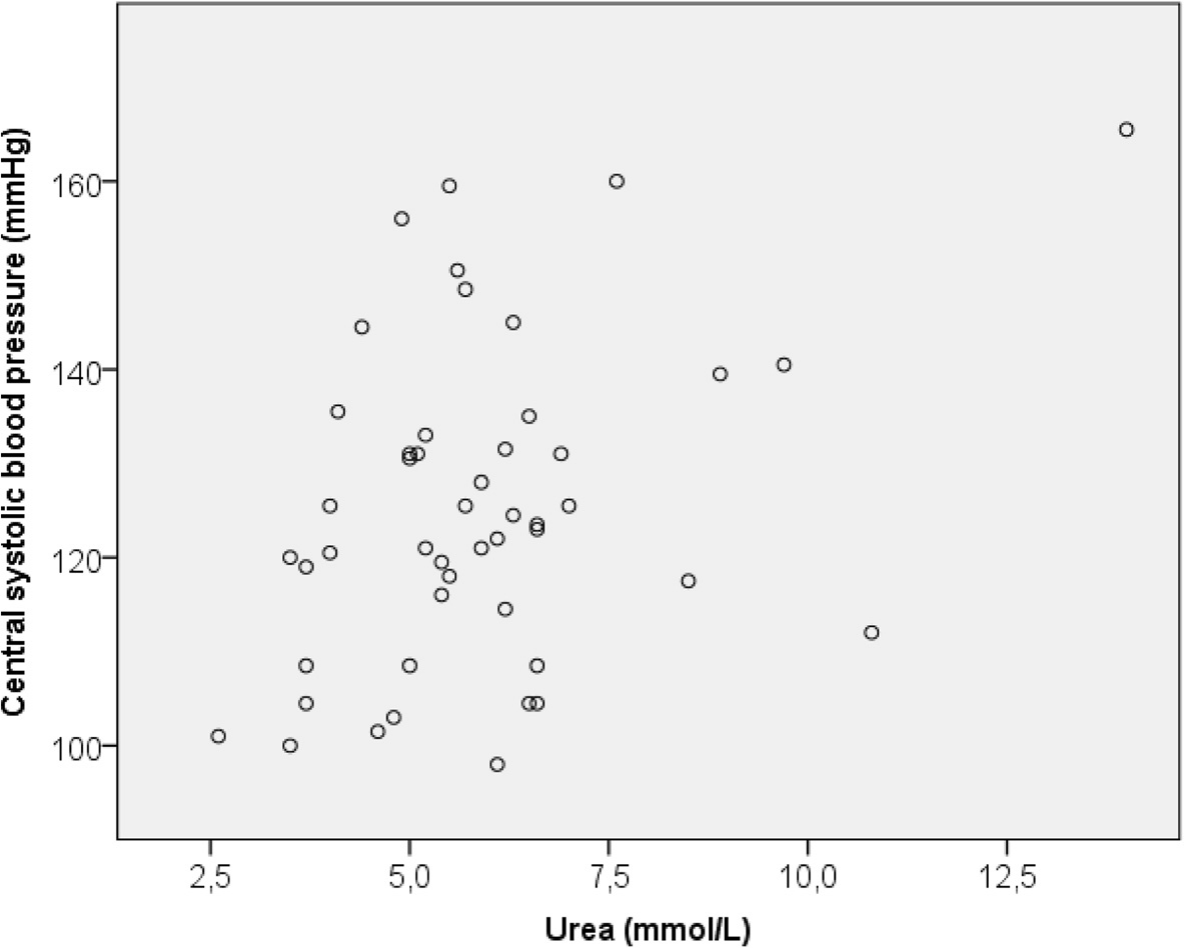
The association between central systolic pressure and serum urea level (rho = 0.318, *p* = 0.029)

In the OA patients, serum urea level correlated positively with car-fem PWV (Fig. 1) and central systolic blood pressure (Fig. 2). There were no associations between parameters of arterial stiffness and urea in the control group. In multiple regression analysis (Table 3), where car-fem PWV was set as the dependent variable, age (*p* < 0.001), mean arterial blood pressure (*p* = <0.001) and OA status (*p* = 0.029) were found to be independent predictors while urea was not significant (*p* = 0.233).

**Table 3:** Multiple regression analysis with carotid-femoral pulse wave velocity as dependent variable (adjusted R^2^ = 0.48), *n* = 97

	Regression coefficient	β	SE	*p*-value
Constant	−6.68		1.81	<0.001
Age	0.15	0.53	0.02	<0.001
Mean pressure	0.07	0.36	0.01	<0.001
OA status	0.74	0.17	0.33	0.029
Urea	−1.20	−0.09	0.10	0.233

## Discussion

The results of this case-control study demonstrate that OA patients have increased central arterial stiffness compared to non-OA controls. The study gives a comprehensive overview of arterial stiffness measured, with the use of different validated methods in end-stage OA patients and controls. The study used both patient related outcome measure and (SF36) and physician related outcome measures. To the best of our knowledge, this is the first study to demonstrate increased level of car-fem PWV- gold standard measure of arterial stiffness- in hip and knee OA patients.

Stiffening of the arteries causes an increase in systolic and a decrease in diastolic pressure [28]. Rise in systolic pressure increases the cardiac afterload while lower diastolic pressure reduces coronary perfusion [29]. Arterial stiffness is one of the main determinants of cardiovascular risk and has been found to be an independent risk factor for the development of CVD and increased mortality in many epidemiological studies [30–32]. Implementation of arterial stiffness assessment into clinical practice helps identify patients with higher cardiovascular risk [32]. Car-fem PWV is the gold standard method for quantifying arterial stiffness and has been accepted as an intermediate endpoint for cardiovascular events [8]. The AIx is a parameter that mainly describes wave reflections [33]. In healthy vessels the reflected waves augment diastolic pressure in the aortic root and hence increase coronary blood flow; however, in the case of stiffened arteries, waves arrive earlier in systole and increase end-systolic pressure [33]. Measurements of central blood pressure have been found to be a better predictor of future cardiovascular events than brachial blood pressure [34]. Furthermore, there is evidence that central blood pressure is more closely related to end-organ damage than peripheral blood pressure [35–37]. The C2 describes primarily wave reflections that are produced by small arteries and branching points [37] and has been shown to independently predict mortality and future cardiovascular events [31, 38].

In our study we found significantly higher car-fem PWV in the OA patients compared to the apparently healthy controls of approximately the same age and with similar BMI. Since there were no differences in blood pressure or the proportion of hypertensive subjects, the effect of hypertension on arterial stiffness was similar in both study groups. Arterial stiffness parameters have rarely been studied in OA patients. Saleh et al. [9] found association between hand OA and car-fem PWV but it was largely attributable to the confounding effect of age. Goldsmith et al. [10] found no difference in arterial stiffness between subjects with and without knee bone marrow lesions. The discrepancy between the above results and those of the current study could be explained by the fact that our patients had a more advanced stage of OA. Due to pain and functional disability, the level of physical activity is limited in the advanced stages of OA. Since physical activity is associated with arterial stiffness [39], increased pain and higher functional disability status might explain increased arterial stiffness in OA patients. In support of our hypothesis, a recent study has also found elevated stiffness of the aorta in end-stage OA patients [40]. Many factors, such as reduced physical activity, inflammation and oxidative stress, which are present across end-stage OA patients might be responsible for the stiffer arteries. Obesity is a known risk factor of both OA and CVD; Adipokines (white adipose tissue derived hormones) are associated with CVD and have also been shown to play an important role in OA development and progression, which allows to link OA with obesity. Aging is another potential link between OA and CVD. Accumulation of advanced glycation end-products is a feature of aging that is present in both diseases [41, 42].

Although OA has generally been considered a non-inflammatory condition, the role of inflammation is increasingly being recognised [43–45]. We found increased level of hsCRP in OA patients. The hsCRP is a marker of low-grade systemic inflammation and has been shown to increase in OA [43]. In addition, inflammation and elevated levels of hsCRP have been directly associated with arterial stiffness [16] and increased cardiovascular risk [31]. Oxidative stress has also been found to be related to arterial stiffness and endothelial dysfunction [16]. Analogously, there is evidence to suggest that oxidative stress is involved in the pathogenesis of OA by causing chondrocyte senescence and apoptosis [13]. We found a significantly higher level of oxidative stress index in patients with OA compared to healthy controls (paper in preparation), which is in line with previous results and with the hypothesis that oxidative stress might be at least partly responsible for the increased arterial stiffness in OA.

A number of studies have demonstrated higher CVD prevalence among OA patients [4, 6]. One plausible explanation for the association of CVD with OA is that vascular pathology plays a role in development of OA. Vascular damage to the subchondral bone has been proposed as a possible initiator of OA [46]. Blood supply to the subchondral bone region may be disturbed by microemboli and venous stasis. Highly vascularized epiphysis is mainly supplied with blood via the epiphyseal artery, which makes this region of high nutrient demand particularly susceptible to perfusion insufficiencies. In support of this hypothesis, Chang et al. [47] found evidence that osteoblasts and chondrocytes from osteoarthritic joints suffer from hypoxia. Furthermore, they also found that hypoxia induced production of matrix metalloproteinase 9 and proangiogenic factors and caused reduction in osteoblast mineralized bone nodule formation, which are all characteristic of OA*.* Whilst the hypothesis of vascular involvement in OA pathology is gaining support, the potential role of increased arterial stiffness in OA patients’ increased cardiovascular risk remains unclear.

We found car-fem PWV to be positively correlated with urea in OA patients. Since urea is the end-product of amino acid metabolism, this association highlights the possible role of altered amino acid metabolism in OA patients. Changes in amino acid profiles and impaired kidney function have been shown to contribute to increased arterial stiffness [48, 49]. On the basis of eGFR levels, which were approximately the same across both groups in this study, there was no difference in the kidney function. After adjusting for the effects of age and mean pressure, the association between urea and car-fem PWV was no longer significant. Higher applied forces (higher blood pressure) in aorta and aging is a strong independent predictors of car-fem PWV [50]. It is therefore important to adjust for mean pressure and age when evaluating predictors of car-fem PWV. In the present study, the association between urea level and car-fem PWV was largely due to the confounding effects of age and mean arterial blood pressure. Additional analysis exploring the effects of body composition, smoking status, functional status and use of anti inflammatory drugs on arterial stiffness in OA patients should be within the scope of future studies.

This study had several limitations that need to be recognised. First, because of the cross-sectional design of this study, it was not possible to establish causal associations between OA and arterial stiffness. Second, since no radiographs were taken in the control group, some of the controls might have had asymptomatic OA and a type II error might have been introduced. Third, this study had a relatively small sample size and hence limited statistical power; concequently, the results need to be verified by larger studies. Fourth, this study did not account for effects of the body composition, smoking status, functional status or use of anti inflammatory drugs, which might influence arterial stiffness.

## Conclusions

In conclusion, our results provide evidence that patients with end-stage OA have increased central arterial stiffness. Arterial stiffness might be important regarding the development of OA and increased cardiovascular risk. However, causal association between OA and vascular stiffness needs to be confirmed by larger-scale studies.
